# A case report of resected ectopic malignant meningioma with lung metastasis

**DOI:** 10.1097/MD.0000000000015853

**Published:** 2019-06-07

**Authors:** Kotaro Murakami, Hidenobu Takahashi, Tomokazu Omori, Osamu Uchida, Hiroshi Hirano, Norihiko Kawate, Norihiko Ikeda

**Affiliations:** a Department of Thoracic Surgery; b Department of Pathology, Hachioji Medical Center, Tokyo Medical University; c Department of Health Science and Social Welfare, Waseda University School of Human Sciences; d Department of Surgery, Tokyo Medical University, Tokyo, Japan.

**Keywords:** anaplastic meningioma, ectopic malignant meningioma, Ki-67 labeling index, lung metastasis

## Abstract

**Introduction:**

Meningioma is mostly a benign tumor, but sometimes it is malignant, and there have been reports of distant metastases.

**Patient concerns:**

The patient, a woman in her 40s, was under follow-up after resection of an ectopic malignant meningioma originating in the left axilla. She was referred to our department because of a nodule shadow in the right lung on chest computed tomography (CT) 3 years and 5 months postoperatively.

**Diagnosis:**

Chest CT showed a 1.0 cm nodule shadow in the right S4, which was positive on positron emission tomography-CT; no abnormality was found in any other organ. Therefore, it was considered to be a metastatic lung tumor.

**Interventions:**

Right middle lobe partial resection was performed using thoracoscopic surgery, and a diagnosis of pulmonary metastasis of ectopic malignant meningioma was made by histopathology and immunohistochemistry.

**Outcomes:**

In this case, complete resection was possible.

**Conclusion:**

Meningioma occurs mainly in the cranium, and occurrence in the soft tissue of the extremities is extremely rare. To our knowledge, ours is the first report of a histologically malignant ectopic meningioma with metastasis to the thoracic cavity.

## 1 Introduction

Meningioma is the most frequent primary brain tumor and is derived from meningothelial cells in the neural crest. Meningioma occurs primarily in the cranium, but ectopic meningiomas that occur outside the central nervous system are occasionally seen. However, meningiomas occurring in the soft tissues of the trunk and extremities are extremely rare, and only a few cases have been reported. Moreover, malignant meningioma alone has been reported to have an incidence of metastasis of approximately 43%, which is higher than that of all the meningiomas (0.76%).^[1]^ We experienced a case of axillary primary ectopic malignant meningioma, which, following complete resection, developed a lung tumor that was diagnosed as pulmonary metastasis of meningioma.

## 2 Case report

**Figure 1:**
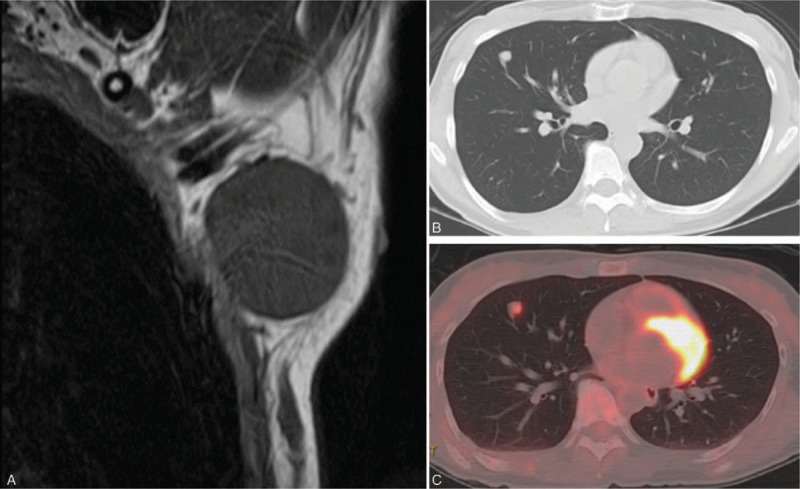
(A) Magnetic resonance imaging revealed a clearly distinct tumor and a low signal in the T1-weighted image. (B) Chest computed tomography showed a 10 × 10 mm nodule in the right S4 area. (C) Positron emission tomography-computed tomography showed slight uptake in the nodule, with a maximal standardized uptake value of 2.94.

**Figure 2:**
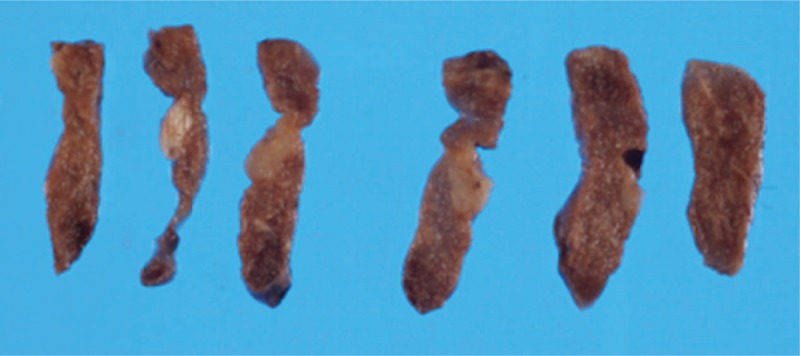
Resected specimen of the S4 of the right lung demonstrating a 13 × 10 × 8 mm white grayish nodule.

A woman in her 40s noticed a left axillary mass in November 2013, and in January 2014 she underwent left axillary tumorectomy for suspected schwannoma at our Department of Plastic and Reconstructive Surgery. The tumor was 5.0 × 6.0 cm, and it was pathologically diagnosed as malignant meningioma (Fig. 1A). As metastasis from a primary site was suspected, imaging examinations, including head and neck magnetic resonance imaging and positron emission tomography (PET)-computed tomography (CT) examination, were carried out, but since all were negative, it was diagnosed as ectopic malignant meningioma of the primary axillary soft tissue. After surgery, 50 Gy radiation was locally administered, and she received follow-up outpatient observation. In June 2016, chest radiography showed a nodular shadow in the right lung, so she was referred to our department. There were no notable findings in the laboratory blood test results. Chest radiography indicated a similar circular nodule shadow, 1.1 × 1.0 cm in size, in the right lower lung field. Chest CT showed the 1.0 × 1.0 cm nodule shadow in the right S4; mediastinal and hilar lymph node enlargement was not observed (Fig. 1B). PET-CT demonstrated slight uptake in the nodule, with a maximal standardized uptake value of 2.94 (Fig. 1C). There was no evidence of distant metastasis. After the initial examination, it was considered to be a metastatic lung tumor, and we decided to perform surgery as there were no abnormal findings in other organs. Surgery was performed in the left lateral decubitus under differential lung ventilation and 3-port thoracoscopy. There were no adhesions or pleural changes, and the tumor was directly under the right S4 pleura. Thoracoscopic right middle lobe partial resection was performed and the tumor was resected. A diagnosis of meningioma was obtained by intraoperative rapid diagnosis. The operation time was 1 hour, and the bleeding volume was 20 mL. Macroscopically, the tumor, resected from the S4 of the right lung, was 1.3 × 1.0 × 0.8 cm in diameter and a white grayish irregular nodule (Fig. 2).

**Figure 3:**
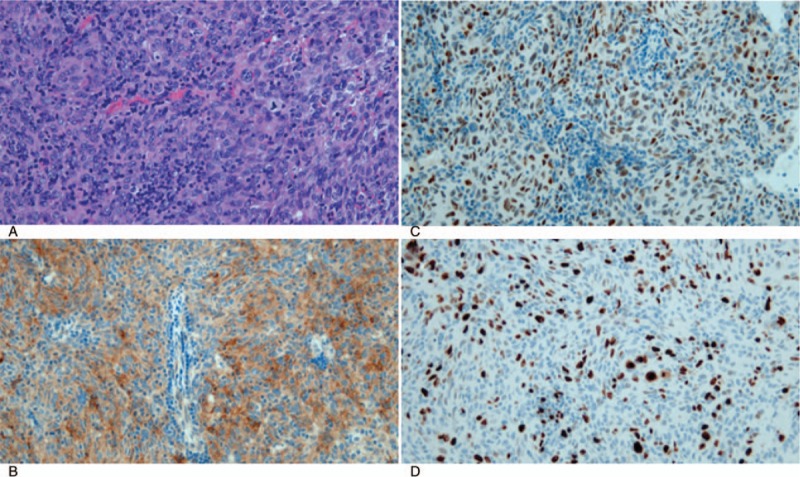
(A) Histopathology showed neoplastic cells with eosinophilic cytoplasm, and coarse chromatin and/or obvious nucleoli. The histological appearance revealed neoplastic cell proliferation with a whorl pattern, mixed with lymphocyte infiltrate (hematoxylin and eosin, ×400). (B) Epithelial membrane antigen expressed on the cell membrane of the tumor cells (×400). (C) Tumor cells expressed the estrogen receptor (×400). (D) The Ki-67 labeling index was approximately 40% in hotspots (×400).

Histologically, the tumor was composed of neoplastic cells with eosinophilic cytoplasm, coarse chromatin, and obvious nucleoli. The histological appearance revealed neoplastic cell proliferation with a whorl pattern, mixed with lymphocyte infiltrate (Fig. 3A).

Immunohistochemically, some tumor cells were positive for epithelial membrane antigen (EMA) (Fig. 3B) and estrogen receptor (ER) (Fig. 3C). The Ki-67 labeling index (LI) was about 40% (Fig. 3D). MNF116 and S100 staining were negative. Based on these findings, the lesion was diagnosed as lung metastasis of malignant meningioma of the primary axillary tissue. Her postoperative course was uneventful, and she was discharged on postoperative day 4. She has remained healthy without any recurrence 9 months after the lung resection. Informed written consent was obtained from the patient for publication of this case report and accompanying images.

## 3 Discussion

Meningioma is a mesodermal tumor with a neural crest origin that develops from meningothelial cells covering the arachnoid surface. They are the most frequent primary cerebral tumors, and more than 90% are considered to be benign.^[2]^ They appear most commonly in middle-aged and elderly women, and most are of unknown etiology. Meningioma exhibits various histologic types and is classified into 15 subtypes by morphological characteristics. Most are benign Grade I, and malignant Grades II and III rarely occur. Grade III, which has a bad prognosis and can be classified as rhabdoid meningioma, papillary meningioma, or anaplastic meningioma, is considered to be a sarcoma and accounts for approximately 1.2% of all meningiomas.^[3]^ The histologic type of this case was anaplastic meningioma, which is considered to be the most malignant among Grade III meningiomas. Malignant meningioma alone is associated with an incidence of metastasis of approximately 43%, which is higher than that reported for all the meningiomas (0.76%).^[1]^ The lung (37.2%) is the most frequent site of metastasis, followed by the bone (16.5%) and spine (15.2%); meningioma is rarely thought to metastasize to the extracranial space.^[4]^

In this case, EMA, vimentin, ER, and D2-40 were positive in both the lung lesion and the axillary primary lesion, and there was no difference in expression between the metastasis and the primary tumor (Table 1). The Ki-67 LI is used as an estimate of the proliferation potency of a tumor and correlates with histologic grade and recurrence rate. The Ki-67 LI is about 1% to 4% for benign meningioma and about 10% to 15% for anaplastic meningioma.^[5]^ In this case, it was estimated that the malignancy and recurrence rates were high, as the Ki-67 LIs were 52.3% in the primary lesion and 40% in the metastatic lung lesion. Ectopic meningioma occurring outside the cranium and inside the spinal canal has also been described. Those occurring in the soft tissues of the trunk and extremities are extremely rare, and only a few cases have been reported, including one case of distant metastasis. However, to our knowledge, no previous cases report metastasis to the lung; this case is the first such report.^[6]^

**Table 1:**
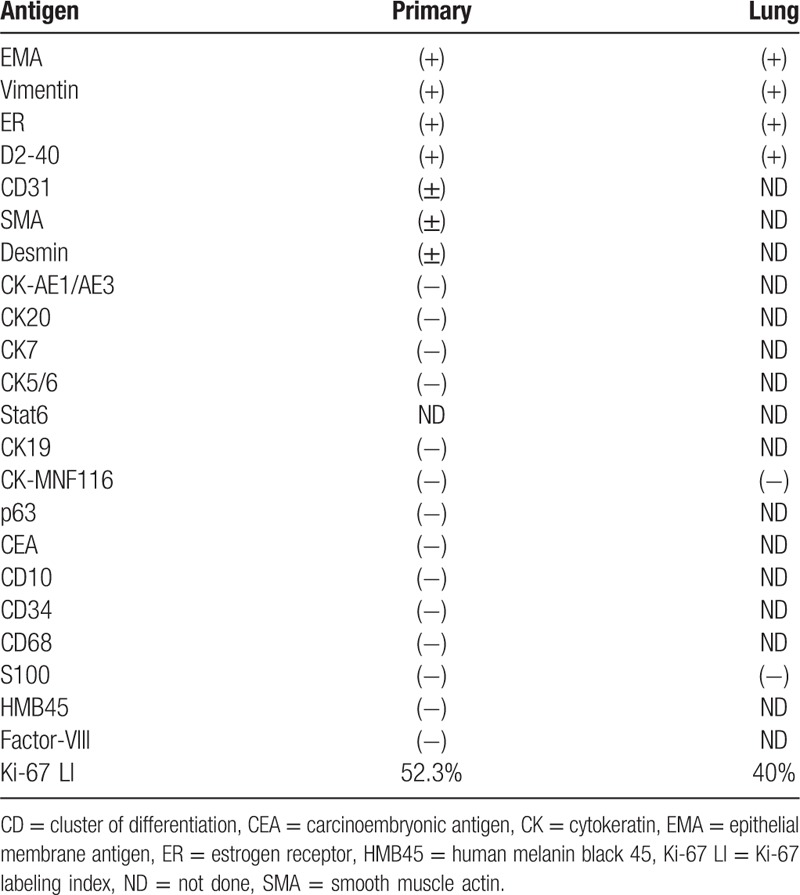
Summary of immunohistochemical study.

There is currently no established treatment for meningiomas exhibiting distant metastasis.

Meningioma is less sensitive to chemotherapy. Radiation therapy is considered effective, but it is ineffective for Grade III meningioma. If it is possible to resect, as in this case, we think that resection should be considered effective treatment. As the Ki-67 LI shows, this case had a high proliferative ability and malignancy, so it is necessary to follow up with careful attention to future recurrence.

## Acknowledgments

We would like to thank Editage (www.editage.jp) for English language editing.

## Author contributions

**Investigation:** Kotaro Murakami.

**Project administration:** Hidenobu Takahashi.

**Supervision:** Norihiko Ikeda.

**Validation:** Hiroshi Hirano.

**Writing – original draft:** Kotaro Murakami.

**Writing – review & editing:** Hidenobu Takahashi, Tomokazu Omori, Osamu Uchida, Norihiko Kawate.
